# Southern marsh deer (*Blastocerus dichotomus*) populations assessed using Amplicon Sequencing on fecal samples

**DOI:** 10.1038/s41598-024-67062-1

**Published:** 2024-07-13

**Authors:** Laura I. Wolfenson, Javier A. Pereira, Daniel E. Ruzzante, Antonio M. Solé-Cava, Gregory R. McCracken, María J. Gómez-Fernández, María D. Pereyra, Patricia M. Mirol

**Affiliations:** 1 División de Mastozoología, Museo Argentino de Ciencias Naturales, “Bernardino Rivadavia”, Av. Ángel Gallardo 470, Ciudad Autónoma de Buenos Aires, CP 1405 Buenos Airesss, Argentina; 2 Department of Biology, Dalhousie University, Halifax, NS Canada; 3 Departamento de Genetica, Centro Nacional Para a Identificação Molecular do Pescado (CENIMP), Instituto de Biologia, Universidade Federal do Rio de Janeiro, Rio de Janeiro, RJ Brazil

**Keywords:** Ecology, Evolution, Genetics, Ecology

## Abstract

Populations in isolated and small fragments lose genetic variability very fast and are usually of conservation concern because they are at greater risk of local extinction. The largest native deer in South America, *Blastocerus dichotomus* (Illiger, 1815)*,* is a Vulnerable species according to the IUCN categorization, which inhabits tropical and subtropical swampy areas. In Argentina, its presence has been restricted to four isolated fragments. Here we examine the genetic diversity and differentiation among three of them, including the three different patches that form the southernmost population, using 18 microsatellite markers genotyped by Amplicon Sequencing of DNA extracted from fecal samples. Genetic diversity was low (H_E_ < 0.45) in all three populations studied. We found three genetic clusters compatible with the geographic location of the samples. We also found a metapopulation dynamics that involves the patches that make up the southernmost population, with evidence of a barrier to gene flow between two of them. Our results point to the creation of a corridor as a necessary and urgent management action. This is the first study, at the population level, employing microsatellite genotyping by Amplicon Sequencing with non-invasive samples in an endangered species.

## Introduction

Nowadays, very little has changed since the loss of biodiversity began to be discussed as the sixth mass extinction^1^. Habitat fragmentation is one of the main threats to large mammal conservation, transforming what once was a continuous population into a number of smaller habitat patches^2^. The genetic consequences of habitat fragmentation are relevant for the outcome of the species inhabiting those patches. If they are small and have low gene flow among them, genetic drift and inbreeding produce loss of genetic variability, which in turn results in reduced survival and reproductive capacity of the individuals. As a consequence, these patches are more likely to suffer local extinctions^2^ This scenario is frequently found at the edges of a species distribution: in those areas, populations are normally exposed to unstable environmental conditions and greater habitat fragmentation. Yet, these areas may foster unique genetic adaptations essential for survival in future climates^3^.

South America bears one of the most diverse and complex faunas in the world^4,5^, including 25% of the world´s mammals^6^. Like the rest of the Neotropical area, it is one of the regions that has undergone the greatest anthropogenic alterations during the last two hundred years, including deforestation, replacement of natural grasslands, increase in human population and the number of road networks, intensification of poaching and introduction of invasive species^7^.

Within this region, Argentina contains one third of all South American mammals, many of which have their southernmost distribution range there^8^, hence representing typical range-edge, fragmented populations. This is the case of the marsh deer, *Blastocerus dichotomus* (Illiger, 1815), the largest deer in South America, an elusive species inhabiting tropical and subtropical swampy areas including alluvial plains with flood pulses, well-vegetated lagoons and marshes with reservoirs^9^. Conditioned by the variable levels of water in these ecosystems, they move seasonally and are excellent swimmers, which allow them to cross wide and mighty rivers.

The species has experienced a 65% reduction of its historical range^9^. Its current distribution is limited to fragmented populations in the Amazon basin in Southeastern Peru, Northeastern Bolivia, and Eastern Brazil; in the Pantanal from Brazil, Paraguay and Bolivia; and in the upper basin and Delta of the Paraná River in Argentina^9,10^. The marsh deer is considered extinct in Uruguay^11^ and Vulnerable globally (IUCN; www.iucnredlist.org).

**Figure 1:**
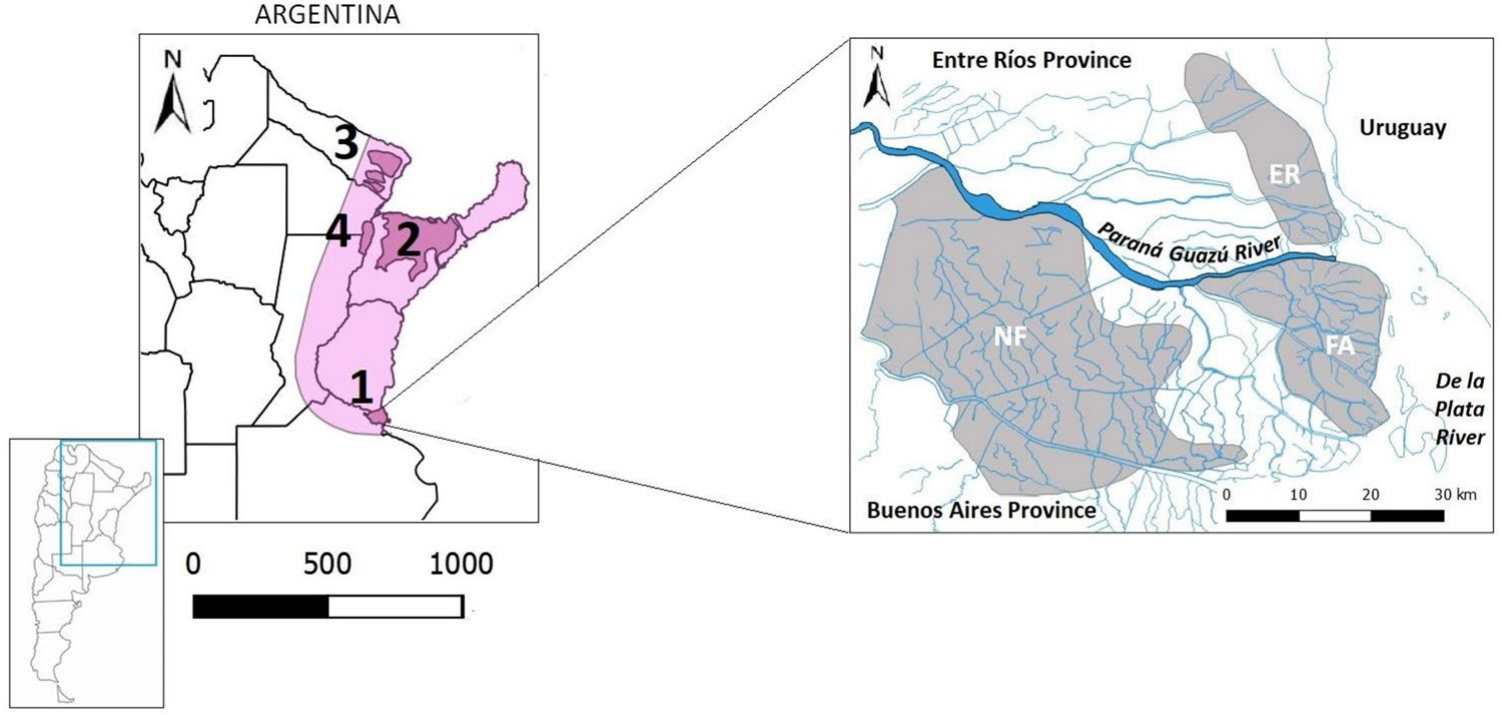
Distribution of *B. dichotomus* in Argentina. Light pink: historical distribution. Dark pink: current populations. 1: Paraná River Delta, divided in three patches (following D’Alessio et al. [53]): Núcleo Forestal (NF), Entre Ríos (ER) and Frente de Avance (FA); 2: Iberá Wetland; 3: Formosa Province (including El Bagual private reserve); 4: Middle Paraná River.

In Argentina, its presence has been restricted to four isolated fragments. Although the species is categorized in the country as Vulnerable^10^, categories vary among the four fragments: Endangered in the Paraná River Delta, Vulnerable in the Iberá Wetland, Endangered in Formosa, and Critically Endangered in the Middle Paraná River (Fig. 1). The main threats in all fragments are habitat degradation and fragmentation, poaching and transmission of diseases from domestic animals^10^.

When studying endangered and elusive species such as the marsh deer, the use of genetic non-invasive sampling (gNIS) is a good alternative to tissue sampling^12^. DNA obtained from non- invasive samples -without capturing, handling or even seeing individuals- can be used for individual identification^13^, estimation of relatedness^14^, census and effective population sizes^15^, sex ratios^16^, inbreeding^17^, genetic variability and structure within or among populations^18^.

Traditionally, gNIS has been combined with microsatellites in molecular ecology studies. The advent of Next Generation Sequencing techniques allowed the use of SNPs, which present a series of advantages over microsatellites, such as requiring less prior laboratory work for their development and optimization, have lower genotyping error rates, require shorter target sequences and produce combinable datasets^19,20^. However, gNIS produce low quality and degraded DNA, a poor source of DNA for SNPs analyses considering the most common and affordable techniques, such as microarrays and RADseq^21^. More recently, the Amplicon Sequencing technique has been developed based on performing massive sequencing of PCR products to obtain genotypes. This technique involves the PCR amplification of a set of *loci*, which are then processed for library preparation adding adapter sequences compatible with the NGS platform. The libraries are then sequenced massively and simultaneously. In the case of microsatellites, the novelty is that results provide not only the different allele sizes, but also their sequences.

The application of Amplicon Sequencing to microsatellite genotyping manages to overcome some of the disadvantages of this kind of markers, such as unambiguous allele identification, information not only on the number of repeats but also on the sequence and comparability among platforms and laboratories^21^. Moreover, it allows the analyses of short target sequences, such as those obtained from gNIS. However, only a few works have sequenced and genotyped microsatellites with Amplicon Sequencing, applied to a low number of non-invasive samples^22–24^.

In this study we examine the genetic variability and connectivity of the remaining habitat patches occupied by the marsh deer in Argentina, focusing especially in the Paraná River Delta, the southernmost edge of the distribution area of the species. To this end, we use microsatellite genotyping by Amplicon Sequencing targeting DNA extracted from non-invasive samples. We aim to provide baseline data for future management actions and conservation of this threatened species.

## Methods

### Sampling and laboratory procedures

We collected fecal samples from the Iberá Wetland, the private reserve El Bagual and the three population fragments (Núcleo Forestal (“NF”), Entre Ríos (“ER”) and Frente de Avance (“FA”)) in the Paraná River Delta (Fig. 1). We couldn´t find samples in the Middle Paraná River, where the species has been categorized as Critically Endangered^10^. For the collection of scats, we surveyed deer trails in areas where the presence of the species had been previously recorded. When feces were found, their location was registered with a GPS coordinate mark and the sample kept in 96% alcohol. Sample vials were stored at − 20 °C for long term storage. Marsh deer is sympatric with spotted deer (*Axis axis*), an invasive spcies, in the Paraná River Delta and in the Iberá Wetland, and with brown brocket (*Subulo gouazoubira*) in the Iberá Wetland and Formosa (Fig. 1). We included known samples (tissues) of both species in the sequencing runs in order to identify the scats corresponding with our target species.

Additionally, blood samples or other tissues were included from marsh deer found dead in the field by local residents, confiscated from poaching events, rescued when found injured or captured for research^25^.

DNA from tissue samples was extracted using the salting out protocol described by Miller et al.^26^, based on the precipitation of DNA using a salt solution and subsequent cleaning of pellets with alcohol at low temperature. For the fecal samples, we used the digestion protocol indicated in Costa et al.^27^, which consists of a double digestion and double purification process and removal of inhibitors. All extractions were carried out in a room separated from PCR and post-PCR procedures using the Quick-DNA Fecal kit (Zymo Research), following the manufacturer´s protocol. We extracted DNA from 374 samples: 258 from the Paraná River Delta (NF: 142, ER: 49, FA: 67), 76 from the Iberá Wetland and 40 from El Bagual. Genotypes were obtained using the 25 markers described in Wolfenson et al*.*^22^, using the exact same protocols, taken from Zhan et al.^28^. First we ran serial dilutions of DNA in 2% agarose gels and selected a 1:50 concentration as template for PCR reactions. The cycling program consisted of 94 °C for 15 min, 20 cycles at 94 °C for 30 s, 57 °C for 180 s, 72 °C for 60 s, and a final extension at 68 °C for 30 min, in a final volume of 5 μl per reaction. The final products were diluted 1:20 and used as template for the second PCR, which adds the barcodes to identify individuals, and consists of 2.15 μl of ultrapure water, 0.5 μl 10 × buffer, 0.2 mM of each dNTP, 0.2 μM of indexed oligo, 0.3 μl of diluted PCR product and 0.25 U of TSG DNA polymerase in a final volume of 5 μl per reaction. The cycling program consisted of 95 °C for 120 s, 20 cycles of 95 °C for 20 s, 60 °C for 60 s, 72 °C for 60 s, and a 72 °C final extension for 10 min. We screened all the PCRs, observing DNA bands by electrophoresis, using 2% agarose gels stained with gel green. The resulting amplicons were pooled, purified and sequenced on an Illumina MiSeq. We sequenced the total of 374 samples and used MEGASAT^28^ to demultiplex the data, genotype all the *loci* and generate the histograms of sequence length-frequency distributions. The output was used to discard inefficient markers (positive amplification was obtained in less than 70% of the samples) and select the samples with the best results (no stutter and histogram bars with at least 20 reads of depth).

Samples from other species were also discarded. Overall, 18 markers and 192 samples were selected. Since DNA extracted from feces is of low quality and in low quantity, we used a modification of the multi-tube protocol according to Frantz et al.^29^, in which a series of individual replicas (3 or more) are genotyped in order to obtain a consensus genotype. Therefore, we performed two additional sequencing runs with three replicates of the 192 samples in each run. Replicas were barcoded as if they were different individuals, in order to compare them. Thus, the consensus genotype was obtained from 7 replicas. Finally, samples with more than 50% missing data were excluded. This resulted in a dataset of 117 samples.

### Error estimation and probability of identity

We used GIMLET v1.3^30^ to evaluate allelic dropout and false allele rates and to identify repeated genotypes, and MICROCHECKER v2.2^31^ to identify null allele rates and search for evidence of large allele dropout and stuttering. For the cumulative identity probability curve, we used GENALEX v6.51b2^32^. We established the number of *loci* necessary to discriminate individuals by P(ID)_sib_, with enough *loci* being necessary to obtain an overall identity probability < 0.01^33^.

### Variability and genetic structure in *Argentina*

We used STRUCTURE^34^ for assessing genetic structure. We ran 10 replicas for each k between 1 and 7, without locality as a prior, with a burn in of 1 × 10^6^ iterations and 5 × 10^6^ additional Markov chains. We repeated the analysis using the locality of origin of the sample as prior. The output was considered reliable when the time-series plots of summary statistics (*F*_*ST*_, Dirichlet parameter (α) and posterior probability) reached stability. The optimal number of genetic clusters (k) was obtained using the Puechmaille method^35^ calculated on the STRUCTURE SELECTOR webserver^36^. This is the recommended method when working with unequal sampling among localities. Finally, we used CLUMPAK^37^ for the consensus bar graph of the Q-values (percentage of membership of individuals to genetic groups).

We used ARLEQUIN v3.5^38^ to assess microsatellite fit to Hardy–Weinberg equilibrium, conducted AMOVA, and estimated *F*_*IS*_*,* allelic diversity, pairwise *F*_*ST*_ comparisons, and linkage disequilibria between markers. For the last two metrics, the Bonferroni correction^39^ was applied with α = 0.05. The number of alleles per *locus* and the rarefactioned allelic richness were calculated with HP RARE v1.1^40^.

The effective population size (*N*_*e*_) was estimated with LDNe^41^, only for those localities with sampling size ≥ 50^42^ and that could be considered a closed population^43^. The program was set to “random mating” and the minimum allele frequency thresholds were set to 0.05.

### Dynamics and genetic structure in the Paraná River Delta

To analyse the genetic sub-structuring within the Paraná River Delta, we ran STRUCTURE using samples from NF, ER and FA and the same settings that we used when evaluating the entire dataset. We carried out a spatial interpolation analysis using ALLELES IN SPACE v1.0^44^, in order to assess the existence of barriers to gene flow, using the options “raw genetic distances” and “connection network of pairs”, with a grid of x = 200 and y = 100. We viewed and edited the map with DIVA GIS v7.5^45^. We tested for the presence of isolation by distance using EEMS, which uses the concept of “effective migration”. According to this paradigm, migration is faster or slower in each area depending on the genetic similarities between individuals^46^.

A Spatial Principal Component Analysis (sPCA) was performed with the R package ADEGENET v2.0. We set the “distance by neighborhood” connection network with a range of 0–8 km, taking into account the distance that a deer can travel within its home range (JA Pereira, unpublished data). The presence of global and local structure was evaluated with a Monte Carlo test (randtest function) using 1000 permutations^47^. We saved the first two spatial principal components (sPC) for representative plots of the genetic structure.

To analyze local fragmentation within the Paraná River Delta, we used the “Ecological Paradigm” as a theoretical framework^48^: in the distribution of a species, units are previously defined as “a set of individuals that live in the same patch of habitat and therefore interact with each other”^49^, and they are considered demographically independent when the migration rate (m) between them, measured as the proportion of a unit that comes from another unit, falls below 0.1^48^. We used BAYESASS^50^ to estimate migration rates between pairs of previously defined units (NF, ER and FA, Fig. 1). We ran 5 × 10^6^ iterations with 1 × 10^6^ for burn in and thinning intervals of 1000. The mixing parameters of migration rates (m = 0.36), allelic frequencies (a = 0.35) and inbreeding coefficient (f = 0.4) were set following the program’s manual. Convergence was checked with TRACER v1.7^51^.

### Ethical approval

All samples were collected and transported according to the following permits and protocols: Research Permit by Organismo Provincial para el Desarrollo Sostenible (Disp.no. 068/16), Authorization 011/15 by Dirección General de Recursos Naturales de la provincia de Entre Ríos, Proyecto NEA N° 488 by Administración de Parques Nacionales, Exportation Permit N° 044869 (Argentina) and Importation Permit N° 19CA04350/CWHQ (Canada) by the Convention on International Trade in Endangered Species of Wild Fauna and Flora, and Exportation Permit N° IF-2019-95678169-APN-DNBI#SGP by Dirección Nacional de Biodiversidad (Ministerio de Ambiente y Desarrollo Sustentable).

## Results

### Error estimation and probability of identity

Considering the three sequencing runs, total reads varied from 22 to 25 million and coverage per marker per sample varied between 10 and 750 reads. Our total data set consisted of N = 117 samples (26 tissues and 85 fecal samples) because we kept only those samples that had at least 50% complete data after building the consensus genotype profile. The presence of null alleles could not be ruled out for *loci* Bdi30, Bdi51, Bdi57, Bdi59 and Bdi65. The allele dropout rate varied between 0.01 and 0.014, and the false allele rate between 0.01 and 0.20 between replicates, which points to the importance of doing these replicates when working with non-invasive samples. The average positivity rate of the PCRs was 62%. The maximum cumulative probability of identity, ordering the markers from most to less informative, was 6.71 × e^−08^ in the case of P(ID) and 3.68 × e^−04^ in the case of P(ID)_sibs_, indicating that our markers are suitable for the identification at the individual level^33^ (Supplementary Table S1).

### Genetic variability and structure in *Argentina*

No marker deviated from HWE in any of the sampled localities (Supplementary Table S1), therefore all markers were included in the subsequent analyses. No evidence of linkage disequilibrium between markers was found in any of the pairwise comparisons (153 comparisons, all p-values α_Bonferroni_ ≥ 0.0003). Six individuals were resampled in the same locality, thus only one was included in subsequent analyses. Finally, the total number of individuals was 111 (26 tissues and 79 fecal samples) (Table 1). This data set is 86.54% complete (13.46% missing data), and includes 11 individuals from El Bagual, 36 from the Iberá Wetland, and 24, 11, and 29 from each of NF, ER and FA, the three locations in the Paraná River Delta. The mean number of alleles per *locus* was 3.22 (+ */− *1.44). FA had a slightly higher allelic richness than the rest of the localities, while El Bagual was the locality with the lowest allelic richness. FA and El Bagual were the localities with the highest expected heterozygosity. All the localities sampled, including each one of the patches of the Delta, presented private alleles. The highest number of private alleles was found in the Iberá Wetland (Table 1).

**Table 1:** Population genetics statistics for each locality.

Population	N	Ā_n_	A_r_	A_p_	D	H_O_	H_E_
Paraná Delta	64	2.72	2.64	4	0.30	0.37	0.42
NF	29	2.44	2.21	1	0.32	0.35	0.4
ER	11	2.22	2.19	1	0.29	0.4	0.42
FA	24	2.50	2.31	2	0.27	0.4	0.43
Iberá Wetland	36	2.67	2.24	6	0.26	0.38	0.41
El Bagual	11	2.06	2	3	0.32	0.43	0.43

*N* Number of individuals, *Ā*_*n*_ Net number of alleles per average *locus*, *A*_*r*_ Rarefied allelic richness, *A*_*p*_ Number of private alleles, *D* Allelic diversity, *H*_*o*_ Observed heterozygosity, *H*_*E*_ Expected heterozygosity.

**Figure 2:**
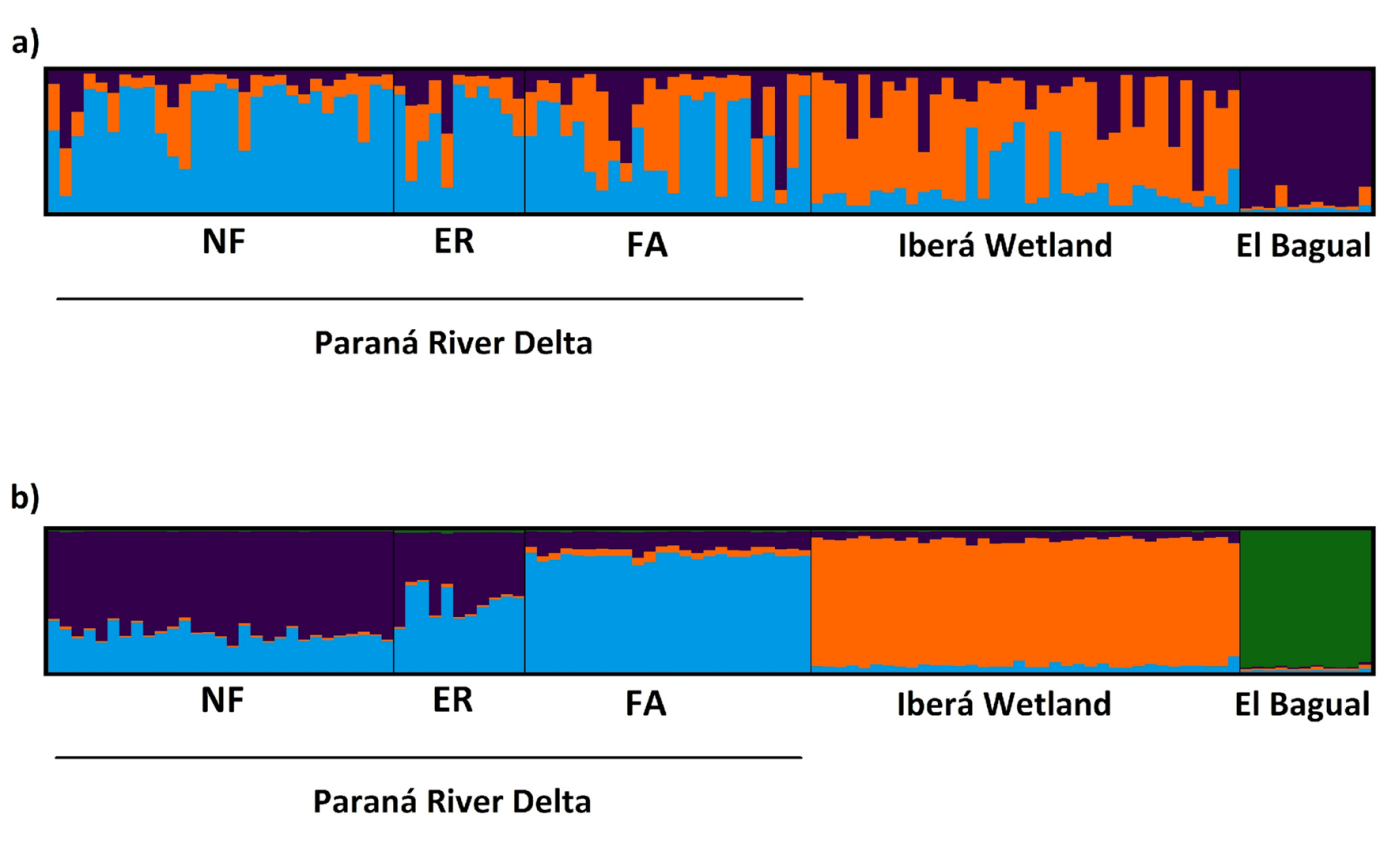
STRUCTURE output including all sampling localities. (**a**) Without sampling location as a prior, the primary structure shows an optimal k = 3. (**b**) Including the sampling location as a prior, there is a secondary structure with an optimal k = 4. Note that the current study does not include collections from the Middle Paraná.

The STRUCTURE analysis without the locality as a prior detected an optimal k of three: the Paraná River Delta (including its three patches), the Iberá Wetland and El Bagual (Fig. 2a). The mixing pattern reveals recent gene flow between the Iberá Wetland and the Paraná River Delta, and a greater differentiation for El Bagual. Independent runs for each population did not find any substructure. However, when using the locality as a prior, the optimal k increased to four, revealing a substructure pattern, clearly separating FA from NF. Individuals in ER show mixed membership between FA and NF (Fig. 2b). Mean membership of ER was 0.57 to the NF cluster and 0.40 to the FA cluster.

The Analysis of Molecular Variance between the five sampling localities found significant differences between them (Supplementary Table S2, ${\widehat{F}}_{ST}$=0.07, *p* < 0.05) Within the Paraná River Delta, pairwise comparisons between patches were contradictory, as there were no significant differences between NF and ER or between FA and ER, but FA was found to be significantly different from NF (Supplementary Table S3). When four localities were considered (grouping ER with FA or with NF alternatively), the NF + ER configuration was the one that minimized the intra-group variance and maximized the between-group variance (Supplementary Tables S4and S5), and all pairwise *F*_*ST*_ comparison where significant (Supplementary Tables, S6). Finally, no locality showed evidence of inbreeding according to the *F*_*IS*_ indices (p(NF) = 0.19; p(ER) = 0.8; p(FA) = 0.99; p(Iberá) = 0.95; p(Bagual) = 0.75).

### Dynamic and genetic structure in the Paraná River Delta

**Figure 3:**
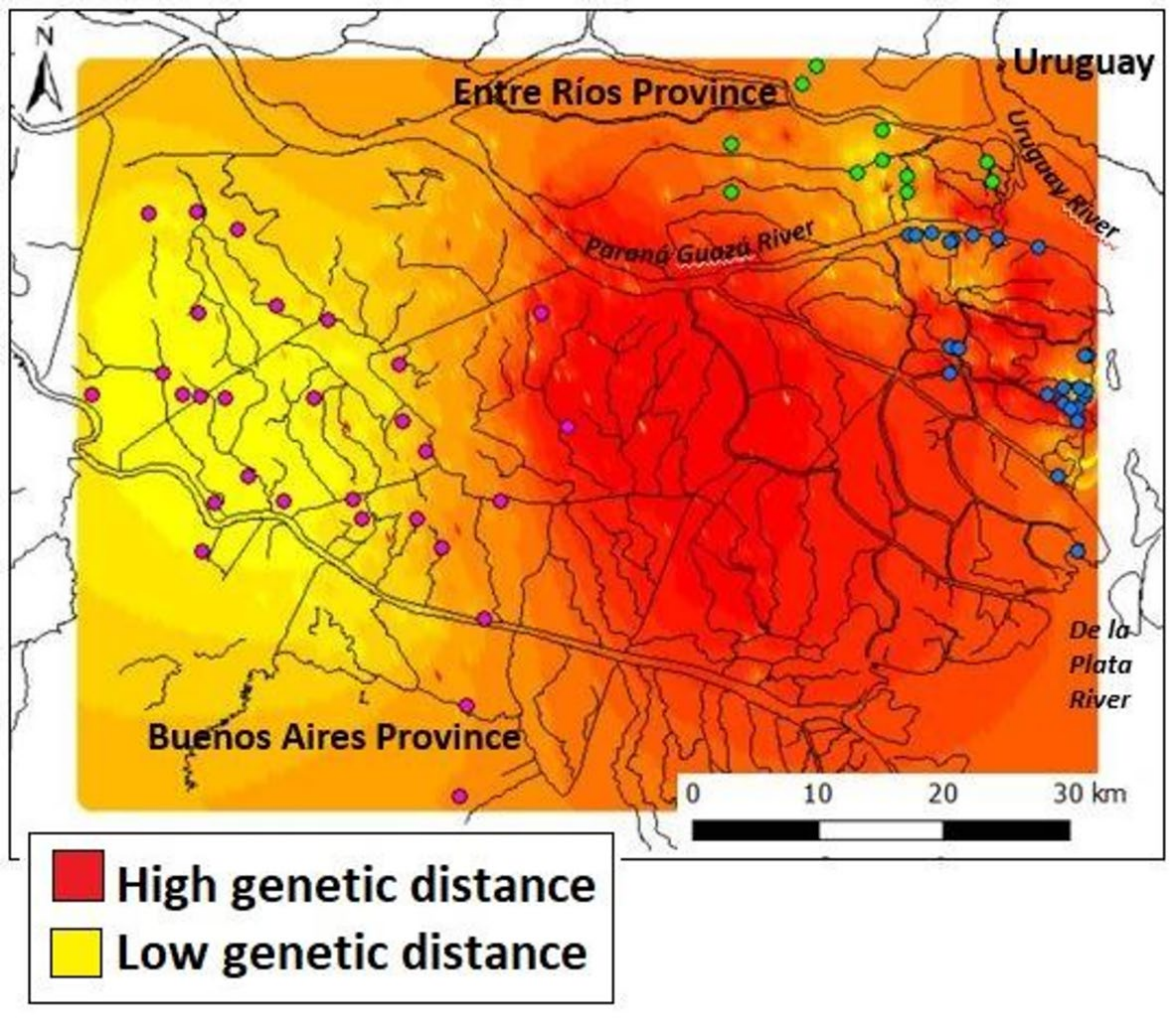
Spatial interpolation Analysis in the Paraná River Delta. Dots represent samples: pink: NF, green: ER, blue: FA. The red zone between NF and FA indicates the presence of a barrier to gene flow.

The spatial interpolation analysis on the Delta population resulted in areas of different genetic distance between individuals (Fig. 3). NF individuals appear mostly in a zone of low genetic differentiation, while FA individuals are in a zone of high genetic differentiation. The area between FA and NF (where there are no individuals or the population density is extremely low^52^) indicates a marked change in genetic similarity, suggesting a barrier to gene flow. We didn’t find any evidence that there’s an isolation by distance pattern in the area (R = 0.07, *p* > 0.05).

**Figure 4:**
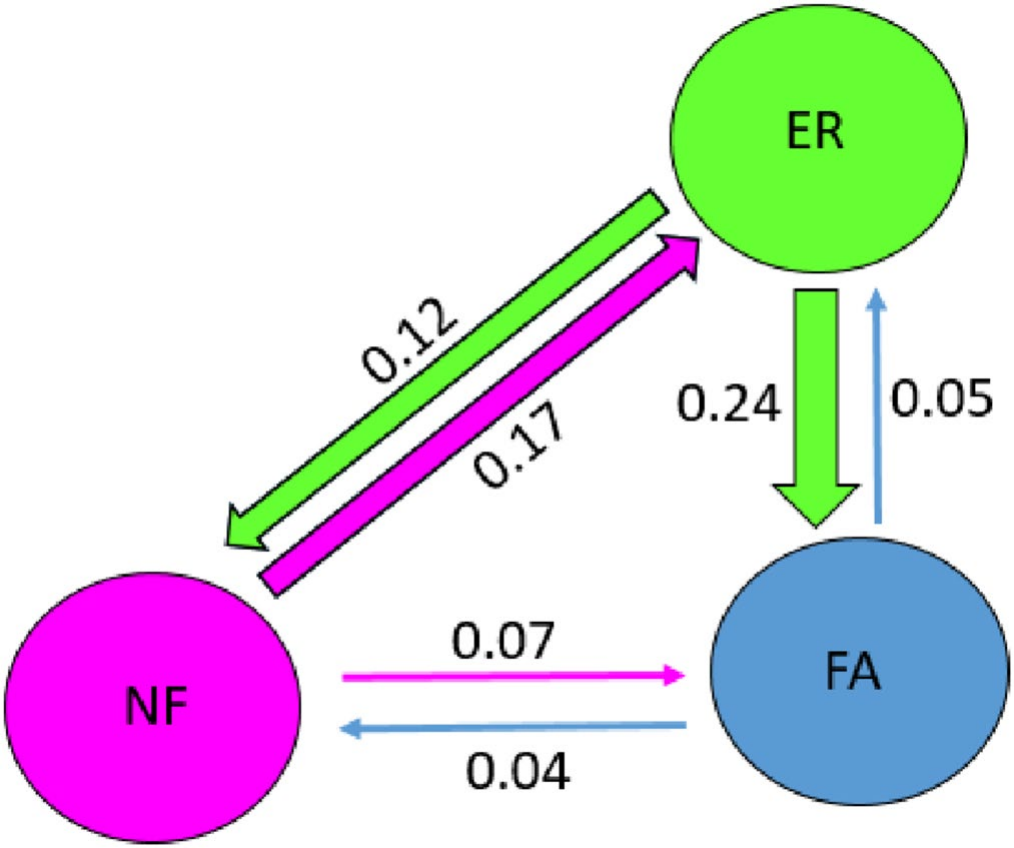
Migration rates between patches in the Paraná River Delta. Numbers above arrows indicate the mean proportion of individuals that migrate between patches.

Migration rates place ER as an area of connection between NF and FA (Fig. 4). Direct migration between these last two patches is low [m < 0.1). Surprisingly, migration between FA and ER, across the Paraná Guazú River (the largest of the arms of the Paraná River Delta), seems to occur almost exclusively from the latter to the former.

Monte Carlo tests in sPCA analysis indicated structure at the global but not at the local scale (Robs = 0.35, *p*-value ≤ 0.02; Robs = 0.34, *p*-value ≥ 0.24). The first two sPCs were retained for interpretation (Supplementary Fig. S1). The first sPC (11.13% of the total variance) revealed a structure that separates NF from FA + ER. The second sPC did not add any useful information and is not displayed.

The effective population size for the Paraná River Delta three patches (NF, ER, FA) pooled was ${\widehat{N}}_{e}$= 44.1 ± 25.8. El Bagual and the Iberá Wetland were excluded from the analysis for having less than 50 samples.

## Discussion

### Genetic structure and variability

The genetic variability indices (H_E_: 0.41–0.43, Table 1) were low and similar among all localities, well below the heterozygosity values reported for the population of *B. dichotomus* from the Brazilian Pantanal, a population in a better conservation status (H_E_: 0.77)^52^. This is likely related to a drastic reduction in population sizes and then a rapid and recent growth such as that recorded in the three populations studied after the creation of protected areas^10,53^. The structure analysis showed an admixture pattern in the Paraná River Delta and the Iberá Wetland, suggesting relatively recent gene flow among them—that is, in the order of the last few hundred years- vindicating the value of riparian environments in large rivers as biological corridors. The westernmost population of the species in Argentina, El Bagual, constitutes a homogeneous genetic group, with the majority of its individuals with high membership coefficient to one cluster. The distance between El Bagual and the Iberá Wetland is approximately 250 km, while the latter is located approximately 700 km from the Paraná River Delta. It is striking, then, that the patches of the Delta and the Iberá Wetland, with more than twice the geographical distance between them than between the Iberá Wetland and El Bagual, show evidence of recent connection. One potential explanation for this outcome could be based on the assumption that, following their isolation from each other, El Bagual population experienced the most significant decline due to factors such as hunting and environmental degradation. As a result, it may have undergone the most severe bottleneck among the three populations studied. The low allelic richness found in El Bagual would be supporting this hypothesis. Another possible non-exclusive explanation is that connection among populations was in the past mainly maintained through riverine margins (which connect the Iberá Wetland with the Paraná River Delta) and more discreetly across the Paraná River (that separates the Iberá Wetland and El Bagual).

Within the Paraná River Delta, there is a substructure pattern in which it is not entirely clear whether ER should be grouped with FA or with NF (Fig. 2b). The ALLELES IN SPACE map (Fig. 3) shows that the genetic distance between the individuals of ER and the other two localities (orange areas) is smaller compared to that between NF and FA. In addition, there is an area identified as a barrier to gene flow between FA and NF (Fig. 3, red area without individuals). Furthermore, the BAYESASS analysis (Fig. 4) shows considerable migration rates between ER and the other two localities. Altogether, it seems that ER functions as a connecting zone for migration between NF and FA.

The sPCA plot (Supplementary Fig. S1) showed a structure pattern that is consistent with FA and ER belonging to the same group, different from NF. According to the ecological approach described in Waples and Gaggiotti^48^, NF and FA are potentially two demographically independent units. However, as migration is significant at least between some of the three units, the Paraná River Delta could be defined as a metapopulation. Further studies would be needed to support this definition.

These results indicate the need for the creation of a corridor between NF and FA as a necessary and urgent management action for the conservation of the Paraná River Delta metapopulation. The suggested corridor would counteract the gene barrier detected (Fig. 3), which is possibly associated with an artificial water channel constructed approximately one hundred years ago, the starting point of an accelerated anthropization of the region. It is also of the utmost importance to increase the protection of the ER patch, because it is at present acting as the only connection between FA and NF.

A study of movement patterns of marsh deer using radio collars is currently underway in the Paraná Delta (JA Pereira, unpublished) and will complement the results of this study for the elaboration of strategies to prevent or reverse the increase in isolation among patches. Several studies show that, within ungulates, many species use different routes depending on the season of the year, so the challenge of conserving them can be particularly complex because it means managing large areas^54^.

### IUCN categories

Willoughby et al.^55^ studied whether there is an association between the criteria established by the IUCN (www.iucnredlist.org) to categorize the conservation rank of vertebrate species and the most basic measures of genetic diversity used in Conservation Genetics: heterozygosity and allelic richness. The IUCN criteria “extent of the species’ distribution range” and “number of mature individuals” proved not to be predictive of genetic diversity within any of the vertebrate classes. The “reduction in population size” criterion served as a predictor of genetic diversity only in mammals and amphibians, but not in the rest of the vertebrate classes. Taken together, these results suggest that species with low genetic diversity are being overlooked by the current IUCN methodology, except in cases where the population has undergone a drastic reduction in population size. The authors suggested the incorporation of a new criterion with the capacity to identify species at risk due to low genetic diversity, using effective population size and heterozygosity to estimate the number of generations (t) needed for heterozygosity to fall below 0.54 (see^55^ for details). Thus, when t ≤ 10, the species is Critically Endangered; t ≤ 50, Endangered and t ≤ 100, Threatened.

In the case of the marsh deer in the Paraná River Delta, with H_o_ = 0.37, the metapopulation would be classified as “Critically Endangered”. This differs from the assessment of this population made by the Argentine Society for the Study of Mammals^10^, which categorized it as “Endangered”. This proposed genetic criterion has not been yet adopted by IUCN, however, we believe that is a valid alternative to contemplate genetic variability when ranking conservation priorities. The case of *B. dichotomus* in the Paraná River Delta is an example of discrepancies between what is observed using genetic data (e.g.: this work) and ecological data (e.g.: IUCN´s categories), indicating the need to pay attention to both fields when making decisions.

### Effective population size

Effective population size generally provides a good estimate of the rate of loss of genetic diversity in closed populations^43^. The effective size estimated here was low (${\widehat{N}}_{e}$=44 ± 25.8). However, this estimate must be analyzed carefully, since ${\widehat{N}}_{e}$ is influenced by many factors that can produce biases^42,43^. Some of these factors are very common and almost unavoidable when working with wild mammal populations, such as overlapping generations or differences in reproductive success.

On the other hand, expected heterozygosity is reduced in structured populations (Wahlund effect,^56^), which can produce an increase in the linkage disequilibrium signal, and this biases the effective size. Under realistic scenarios, the estimated metapopulation ${\widehat{N}}_{e}$ is expected to be substantially lower than the real one^43^. Furthermore, when a population has undergone recent growth, like in the case of the Paraná River Delta population (JA Pereira, unpublished), ${\widehat{N}}_{e}$ may also be underestimated for a few generations, with the duration and magnitude of the bias proportional to the severity of the bottleneck they have passed through, as the past disequilibrium signal is stronger than the current one^42^. Finally, it must be taken into account that when the sample size is less than 2N_e_, there is a default bias in the estimation^42^.

All this implies that the estimation will be much better when the researcher has both knowledge of the biology of the species (mating system, male/female ratio, etc.) and a certain prior idea of the census size, which allows making informed assumptions about the probable N_e_/N relationship. In the case of this work, at the time of sampling there was not enough information to make these approximations. However, some valuable conclusions can be drawn.

A recent study has collected information on deer abundance in the Paraná River Delta. The use of drones in a forestry establishment of 113 km^2^ in NF, revealed the presence of between 559 and 908 individuals of marsh deer^57^. Palstra and Ruzzante^43^ averaged the N_e_/N ratio of threatened species, obtaining a value of 0.36. Bearing this in mind, the ${\widehat{N}}_{e}$ obtained here is surely quite underestimated, because the N_e_/N ratio considering only the individuals found in that property (which represents only 4.2% of the extension area of presence of the species in Paraná River Delta), is an order of magnitude lower than that average.

It has been established that an N_e_ of 50 is necessary for a population not to be severely affected by inbreeding, and an N_e_ of 500 to maintain its evolutionary potential^58^. The ${\widehat{N}}_{e}$ obtained here is below 50. However, no evidence of inbreeding was found in the population. This also suggests that the value obtained is an underestimation and the real value could be over 50. As mentioned before, population structuring produces a bias by default, which will be greater the greater the differentiation between subpopulations. On the other hand, considering the low genetic diversity observed, it is quite unlikely that the N_e_ is as high as 500. In conclusion, the ${\widehat{N}}_{e}$ value obtained is very low and representative of the current situation of the population, although probably underestimated, surely being in reality a value of 50 < N_e_ <  < 500. This low ${\widehat{N}}_{e}$ is not surprising given it has been observed that, especially within mammals and amphibians, it is very rare that species reach the thresholds of the 50/500 rule^58^. This estimator is also decoupled from the IUCN categories, and its incorporation in species listing criteria would make the classifications more accurate^59^.

### Microsatellites versus SNPs

The present study is the first to use Amplicon Sequencing with non-invasive samples at the population level on a threatened species, constituting a precedent, with good results, for future studies in threatened mammals. At the time of starting the project, only one study had tried the use of Amplicon Sequencing with microsatellites in a low number of non-invasive samples^24^. Later, Eriksson et al.^60^ developed a highly efficient protocol for working with non-invasive samples and this technique but using SNPs, also in a low number of samples. Its strength lies in the use of two purifications with magnetic beads, one after each PCR in the library procedure, with which an efficiency greater than 90% is achieved. Although we did not use Eriksson et al.’s protocol^60^, the data set from the three sequencing libraries we obtained, comprising between 3 and 7 positive replicates per sample, resulted in a reliable genetic profile for 111 individuals, with low rates of allelic dropout errors and false alleles.

Currently, the cheapest Next Generation Sequence (NGS) technique used for SNPs that allows working easily with a high number of *loci* is RADseq (Restriction site-associated DNA), which consists of sequencing regions adjacent to restriction enzyme cuts that occur both in coding and non-coding regions of the genome^21^. But this technique cannot be used with low-quality samples, such as non-invasive samples, because digestion of degraded DNA may result in fragments that are too small to generate enough information^21^.

This happens when working with threatened and/or elusive species, from which samples are found by chance in the field, that is, it is a source of DNA that has been exposed to environmental conditions (rain, sun, microorganism, etc.) and consequently in very poor preservation conditions. Arantes et al.^61^ developed an impressive protocol with a hyena species that is currently classified as Least Concern, from which they obtain samples that are directly collected from the animal (fecal mucus and hair). These samples usually contain DNA of relatively good quality, and that is why the authors manage to work with the RADseq technique in this case. But that possibility does not apply in the case of species that are in very low density and/or that are too elusive to obtain samples using that method.

Eriksson’s work is an alternative to the RADseq-gNIS problem, but it could present other limitations in terms of genetic resolution if it were to be extended to the population level, because there is a compromise in the rate between number of markers and number of samples that can be included in a sequencing plate. If it is also taken into account that non-invasive samples require genotyping at least three times to obtain a consensus genotype, the number of markers that could be used would be very low (*e.g.,* in the order of tens or just over 100) and probably insufficient. For example, for species with high dispersal capacity, a minimum of 80 SNPs is needed to detect a low level of differentiation^62^. Furthermore, the use of SNPs requires that the entire genome is already available or that an array panel is already validated^63–65^.

We want to challenge the idea that SNPs are always the best option. Our final conclusion is that whenever it is not possible to use RADseq, as in the case of non-invasive samples of threatened species, the combination of Amplicon Sequencing with microsatellites provides an advantage over the use of SNPs. Therefore, supporting continued microsatellite use when appropriate is essential.

Simultaneously, we must develop protocols for managing genomic data processed with diverse bioinformatic pipelines^66^.

### Supplementary Information

Supplementary Information.

## Data Availability

The data underlying this article is available in *GenBank*: OL998317, OM001701, OM001702, OM001703, OM001704, OM001705, OM001706, OM00170, OM021322, OM021323, OM021324, OM021325, OM021326, OM021327, OM021328, OM021329, OM021330, OM021331, OM021332, OM021333, OM021334, OM021335, OM021336, OM021337, OM021338.
