# Supplementary material for: Southern marsh deer (Blastocerus dichotomus) populations assessed using Amplicon Sequencing on fecal samples
Source: Sci Rep. 2024 Jul 13;14:16169. doi: 10.1038/s41598-024-67062-1 (PMC11246461; doi:10.1038/s41598-024-67062-1)
Supplement: Supplementary file 1 — Supplementary Information. [file 41598_2024_67062_MOESM1_ESM.pdf]

Supporting Information

Southern marsh deer (*Blastocerus dichotomus*) populations assessed using Amplicon Sequencing on fecal samples

Laura I. Wolfenson[1]*, Javier A. Pereira[1], Daniel E. Ruzzante[2], Antonio M. Solé-Cava[3], Gregory R. McCracken[2], María J. Gómez-Fernández[1], María D. Pereyra[1], Patricia M. Mirol[1]

[1]Museo Argentino de Ciencias Naturales, “Bernardino Rivadavia”, División de Mastozoología, Ciudad Autónoma de Buenos Aires, Argentina.
[2]Dalhousie University, Department of Biology, Halifax, Nova Scotia, Canada.
[3]Universidade Federal do Rio de Janeiro, Instituto de Biologia, Departamento de Genetica, Centro Nacional para a Identificação Molecular do Pescado (CENIMP), Rio de Janeiro, RJ, Brazil.

*Corresponding author:

laura.wolfenson@gmail.com

Av. Ángel Gallardo 470 (CP 1405), CABA, Buenos Aires, Argentina.

Supplementary Tables

Table S1. Statistics for each locus. Ho: Observed Heterozygosity, H_E: Expected Heterozygosity, p(H-W): p-values for Hardy-Weinberg equilibrium test for the whole data set, AD: Average Allelic dropout rate between replicas, FA: Average false allele rate between replicas, %PCR+: Average positive PCR rate between replicas, P(ID): Theoretical probability of identity, P(ID)SIB: Probability of identity between siblings.

Locus	n	A	H_O	H_E	p(H-W)	AD	FA	%PCR+	$P_{(ID)}$	$P_{(ID)SIB}$
Bdi4	106	4	0.18	0.20	0.01*	0.05	0.06	0.69	$6.7e^{-01}$	$8.2e^{-01}$
Bdi6	99	2	0.08	0.10	0.21	0.03	0.01	0.65	$8.1e^{-01}$	$9.0e^{-01}$
Bdi10	96	2	0.23	0.25	0.41	0.07	0.07	0.68	$6.0e^{-01}$	$7.8e^{-01}$
Bdi12	91	2	0.37	0.46	0.1	0.10	0.09	0.54	$4.0e^{-01}$	$6.2e^{-01}$
Bdi21	101	2	0.41	0.48	0.15	0.03	0.18	0.62	$3.9e^{-01}$	$6.1e^{-01}$
Bdi24	107	2	0.55	0.50	0.33	0.11	0.13	0.77	$3.8e^{-01}$	$6.0e^{-01}$
Bdi26	93	2	0.37	0.41	0.31	0.01	0.09	0.56	$4.3e^{-01}$	$6.5e^{-01}$
Bdi30	97	4	0.34	0.57	0*	0.03	0.13	0.60	$2.8e^{-01}$	$5.4e^{-01}$
Bdi36	80	3	0.44	0.47	0.76	0.10	0.11	0.47	$3.9e^{-01}$	$6.2e^{-01}$
Bdi37	95	6	0.75	0.74	0.74	0.08	0.20	0.63	$1.1e^{-01}$	$4.1e^{-01}$
Bdi42	105	3	0.60	0.59	0.22	0.14	0.10	0.75	$2.6e^{-01}$	$5.2e^{-01}$
Bdi44	100	3	0.11	0.11	1	0.03	0.04	0.68	$8.1e^{-01}$	$9.0e^{-01}$
Bdi49	104	4	0.45	0.50	0.42	0.10	0.10	0.67	$3.5e^{-01}$	$5.9e^{-01}$
Bdi51	79	4	0.44	0.59	0.01*	0.08	0.21	0.49	$2.3e^{-01}$	$5.2e^{-01}$
Bdi55	95	2	0.15	0.17	0.18	0.04	0.02	0.59	$6.9e^{-01}$	$8.4e^{-01}$
Bdi57	92	3	0.12	0.16	0.02*	0.03	0.04	0.60	$7.0e^{-01}$	$8.4e^{-01}$
Bdi59	94	7	0.44	0.64	0*	0.11	0.12	0.57	$1.9e^{-01}$	$4.8e^{-01}$
Bdi65	95	3	0.28	0.39	0.02*	0.03	0.12	0.62	$4.4e^{-01}$	$6.7e^{-01}$
Mean	96.06	3.22	0.35	0.41	0.10	0.06		0.62	$4.5e^{-01}$	$6.6e^{-01}$
S.D. (+/-)	7.74	1.44	0.18	0.2	0.057	0.04		0.08	0.21	0.15

*Significant values turn non-significant when applying the test to individual populations

Table S2. AMOVA results considering the five locations studied

```
--------------------------
AMOVA design and results :
--------------------------

          Weir, B.S. and Cockerham, C.C. 1984.
          Excoffier, L., Smouse, P., and Quattro, J. 1992.
          Weir, B. S., 1996.
----------------------------------------------------------------------
 Source of                  Sum of      Variance         Percentage
 variation      d.f.        squares     components       of variation
----------------------------------------------------------------------
 Among
 populations      4          43.463       0.20015 Va              7.22

 Among
 individuals
 within
 populations    106         256.668      -0.15191 Vb             -5.48

 Within
 individuals    111         302.500       2.72523 Vc             98.26
----------------------------------------------------------------------
 Total          221         602.631       2.77346
----------------------------------------------------------------------
 Fixation Indices
      FIS :     -0.05903
      FST :      0.07217
      FIT :      0.01739
----------------------------------------------------------------------

Significance tests (1023 permutations)
------------------

Vb and FIS : P(rand. value > obs. value)  =  0.96090
             P(rand. value = obs. value)  =  0.00587
                                P-value  =  0.96676+-0.00494

Va and FST : P(rand. value > obs. value)  =  0.00000
             P(rand. value = obs. value)  =  0.00000
                                P-value  =  0.00000+-0.00000

Vc and FIT : P(rand. value < obs. value)  =  0.47898
             P(rand. value = obs. value)  =  0.02542
                                P-value  =  0.50440+-0.01593
```

Table S3. Pairwise F_{ST} (above diagonal) and respective p-values (below diagonal) between the five localities. *Bonferroni-corrected significance values.

	**NF**	**FA**	**ER**	**Iberá Wetland**	**El Bagual**
**NF**	-	0.03*	0.01	0.07*	0.14*
**FA**	<0.00001	-	0.01	0.03*	0.13*
**ER**	0.20	0.11	-	0.09*	0.17*
**Iberá Wetland**	<0.005	<0.005	<0.005	-	<0.005
**El Bagual**	<0.00001	<0.00001	<0.00001	0.13*	-

Table S4. AMOVA results for four localities considering ER+FA as one locality

AMOVA design and results :

Weir, B.S. and Cockerham, C.C. 1984.
Excoffier, L., Smouse, P., and Quattro, J. 1992.
Weir, B. S., 1996.

Source of variation	d.f.	Sum of squares	Variance components	Percentage of variation
Among populations	3	39.926	0.20491 Va	7.36
Among individuals within populations	107	260.205	-0.14670 Vb	-5.27
Within individuals	111	302.500	2.72523 Vc	97.91
Total	221	602.631	2.78343	

Fixation Indices

FIS : -0.05689
FST : 0.07362
FIT : 0.02091

Significance tests (1023 permutations)

Vb and FIS : P(rand. value > obs. value) = 0.96090
P(rand. value = obs. value) = 0.00391
P-value = 0.96481+-0.00489

Va and FST : P(rand. value > obs. value) = 0.00000
P(rand. value = obs. value) = 0.00000
P-value = 0.00000+-0.00000

Vc and FIT : P(rand. value < obs. value) = 0.52199
P(rand. value = obs. value) = 0.02444
P-value = 0.54643+-0.01549

Table S5. AMOVA results for four localities considering ER+NF as one locality

```
-------------------------
AMOVA design and results :
-------------------------

          Weir, B.S. and Cockerham, C.C. 1984.
          Excoffier, L., Smouse, P., and Quattro, J. 1992.
          Weir, B. S., 1996.
----------------------------------------------------------------------
 Source of                    Sum of      Variance          Percentage
 variation      d.f.          squares     components        of variation
----------------------------------------------------------------------
 Among
 populations      3           39.568        0.20515 Va            7.37

 Among
 individuals
 within
 populations    107          260.563       -0.14503 Vb           -5.21

 Within
 individuals    111          302.500        2.72523 Vc           97.84
----------------------------------------------------------------------
 Total          221          602.631        2.78535
----------------------------------------------------------------------
 Fixation Indices
      FIS :     -0.05621
      FST :      0.07365
      FIT :      0.02159
----------------------------------------------------------------------

Significance tests (1023 permutations)
------------------

Vb and FIS : P(rand. value > obs. value)  =  0.96481
             P(rand. value = obs. value)  =  0.00489
                                 P-value  =  0.96970+-0.00596

Va and FST : P(rand. value > obs. value)  =  0.00000
             P(rand. value = obs. value)  =  0.00000
                                 P-value  =  0.00000+-0.00000

Vc and FIT : P(rand. value < obs. value)  =  0.48289
             P(rand. value = obs. value)  =  0.03617
                                 P-value  =  0.51906+-0.01858
```

Table S6. Pairwise F_{ST} (above diagonal) and respective p-values (below diagonal) Considering ER and NF as a single locality. *Bonferroni-corrected significance values.

	**FA**	**ER-NF**	**Iberá Wetland**	**El Bagual**
**FA**	-	0.02*	0.03*	0.12*
**ER-NF**	<0.00001	-	0.07*	0.14*
**Iberá Wetland**	<0.00001	<0.00001	-	0.12*
**El Bagual**	<0.00001	<0.00001	<0.00001	-

Supplementary Figures

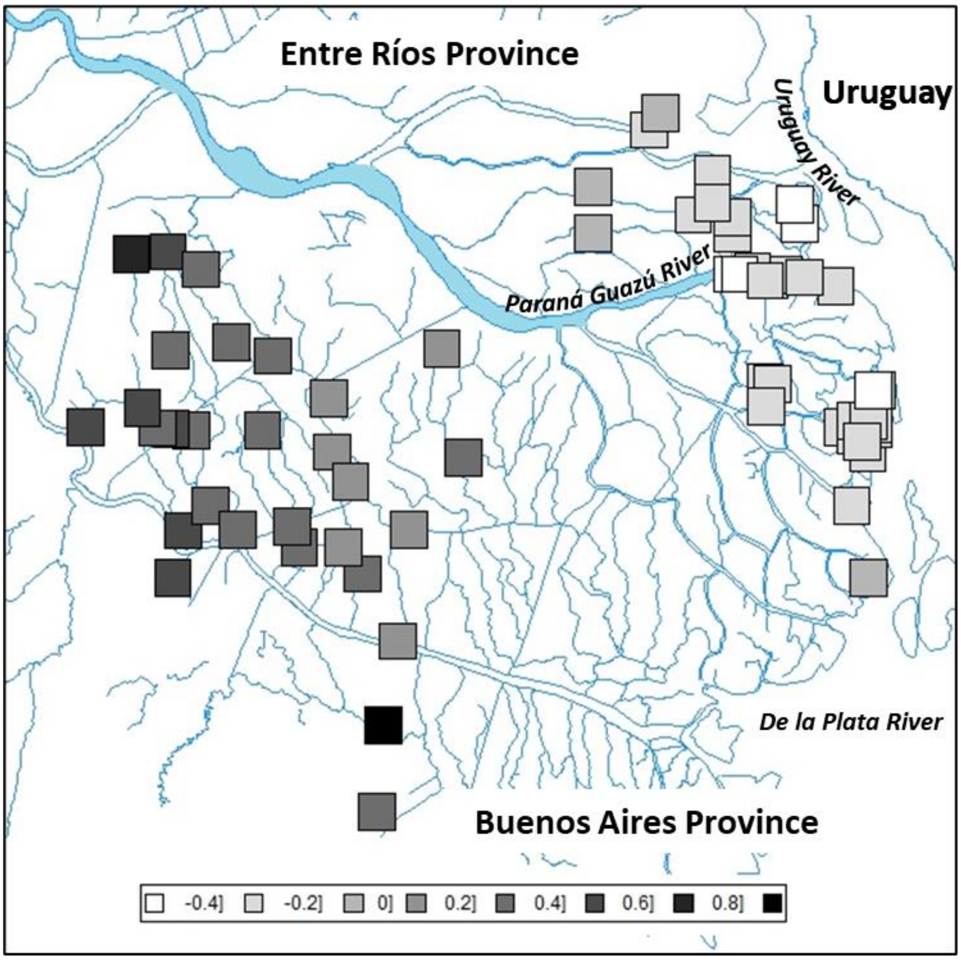

Figure S1. sPCA analysis in the Paraná River Delta. The first sPC plot shows a clear structure pattern between NF individuals (dark squares) and FA+ER (light squares). There is also a soft East-West gradient suggesting isolation by distance.
